# Ocular Inflammation

**DOI:** 10.1155/2015/398076

**Published:** 2015-01-13

**Authors:** Valentín Huerva, Francisco J. Ascaso, Andrzej Grzybowski

**Affiliations:** ^1^Department of Ophthalmology, Universitary Hospital Arnau de Vilanova, 25198 Lleida, Spain; ^2^IRB Lleida, 25198 Lleida, Spain; ^3^Department of Ophthalmology, “Lozano Blesa” University Clinic Hospital, Zaragoza, Spain; ^4^Instituto Aragonés de Ciencias de la Salud, Zaragoza, Spain; ^5^Department of Ophthalmology, Poznań City Hospital, Poznań, Poland

Ocular inflammation has become a hot topic in ophthalmology, involving also several areas of medicine: internal medicine, surgery, basic research, physiology, pharmacology, microbiology, immunology, rheumatology, pharmacology, or laboratory. There are many ocular inflammatory diseases in different locations, including orbit, ocular adnexa, ocular surface, conjunctiva, cornea, sclera, uvea, retinal vessels, and optic nerve. Management of ocular inflammation presents diagnostic and therapeutic challenges, ranging the etiology and prognosis from benign, self-limited conditions to organ-threatening disorders. Ocular inflammation is a component of eye surgery, sometimes leading to its complications, such as macular cystoid edema, and often being targeted by pharmacologic therapy or less invasive surgical procedures. Recently, it has become clear that some assumed to be noninflammatory disorders, like age-related macular degeneration [1] and macular edema secondary to diabetic retinopathy [2] or retinal vein occlusion, are dependent on some inflammatory mediators and thus should be treated, at least partially, as inflammatory disorders.

Ocular inflammation is also often present in patients with systemic inflammatory diseases, such as rheumatoid arthritis, systemic lupus erythematosus, sarcoidosis, Wegener's granulomatosis, Sjögren's syndrome, polyarteritis nodosa, primary antiphospholipid syndrome, Behçet's syndrome, Kawasaki disease, Cogan's syndrome, and relapsing polychondritis. Eye involvement in these conditions can not only be the first symptom of the disease but also serve as a biomarker of the severity of systemic inflammation. Furthermore, immunological therapy is being developed to assist the return of healthy ocular immune responses and immune privilege in the eye.

This special issue reports some pathogenetic, diagnostic, and therapeutic aspects of ocular inflammation. We believe that papers on markers or receptors which play an important role in the pathogenesis of the ocular inflammation deserve special attention. P. Pawlowski et al. showed that macrophages and CD4 T lymphocytes are both engaged in the active, severe, and long stage of inflammation in the orbital tissue of patients with Graves' orbitopathy (GO) in “*Markers of inflammation and fibrosis in the orbital fat/connective tissue of patients with Graves' orbitopathy: clinical implications.*” F. Ekici et al. reported that Eritoran treatment resulted in less inflammatory damage in terms of serum and retinochoroidal tissue parameters in “*Effect of the toll-like receptor 4 antagonist Eritoran on retinochoroidal inflammatory damage in a rat model of endotoxin-induced inflammation.*”

M. Mesquida et al. showed that increased serum levels of IFN-, TNF-, and IL-17A and hsCRP were associated with active uveitis associated with Behçet's disease (BD) and might serve as markers of disease activity in “*Proinflammatory cytokines and C-reactive protein in uveitis associated with Behçet's disease*.” F. J. Ascaso et al. reviewed the role of inflammation in the pathogenesis of macular edema (ME) secondary to retinal vascular diseases. ME is a nonspecific sign of numerous retinal vascular diseases. The role of inflammatory processes in the genesis of both diabetic macular edema (DME) and ME secondary to retinal vein occlusion (RVO) is discussed. The paper discusses the inflammatory mediators which are implicated, the effect of the different intravitreal therapies, the recruitment of leukocytes mediated by adhesion molecules, and the role of retinal Müller glial (RMG) cells.

Finally, another report concludes that, in patients with CMV in the HAART era, immune recovery may be associated with a greater number of inflammatory complications, including ME and epiretinal membrane formation. Given the range of ocular manifestations of HIV, routine ocular examinations and screening for visual loss are recommended in patients with CD4 counts <50 cells/*μ*L in “*Immune recovery uveitis: pathogenesis, clinical symptoms, and treatment*.”

We hope that the readers of this special issue will find accurate data and updated reviews on the mechanisms of the different spectrum of ocular inflammatory disorders. Also, important questions may be resolved, such as inflammatory responses in DME, inflammatory cells in orbital tissues of GO patients, and markers of disease activity in BD cases among others.

*Valentín Huerva* *Valentín Huerva* *Francisco J. Ascaso* *Francisco J. Ascaso* *Andrzej Grzybowski* *Andrzej Grzybowski*
